# Supplementary figures and images for: Transient juvenile demyelination impairs maturation and function of parvalbumin-positive interneurons in the prefrontal cortex
Source: PLoS Biol. 2025 Sep 30;23(9):e3003421. doi: 10.1371/journal.pbio.3003421 (PMC12510662; doi:10.1371/journal.pbio.3003421)

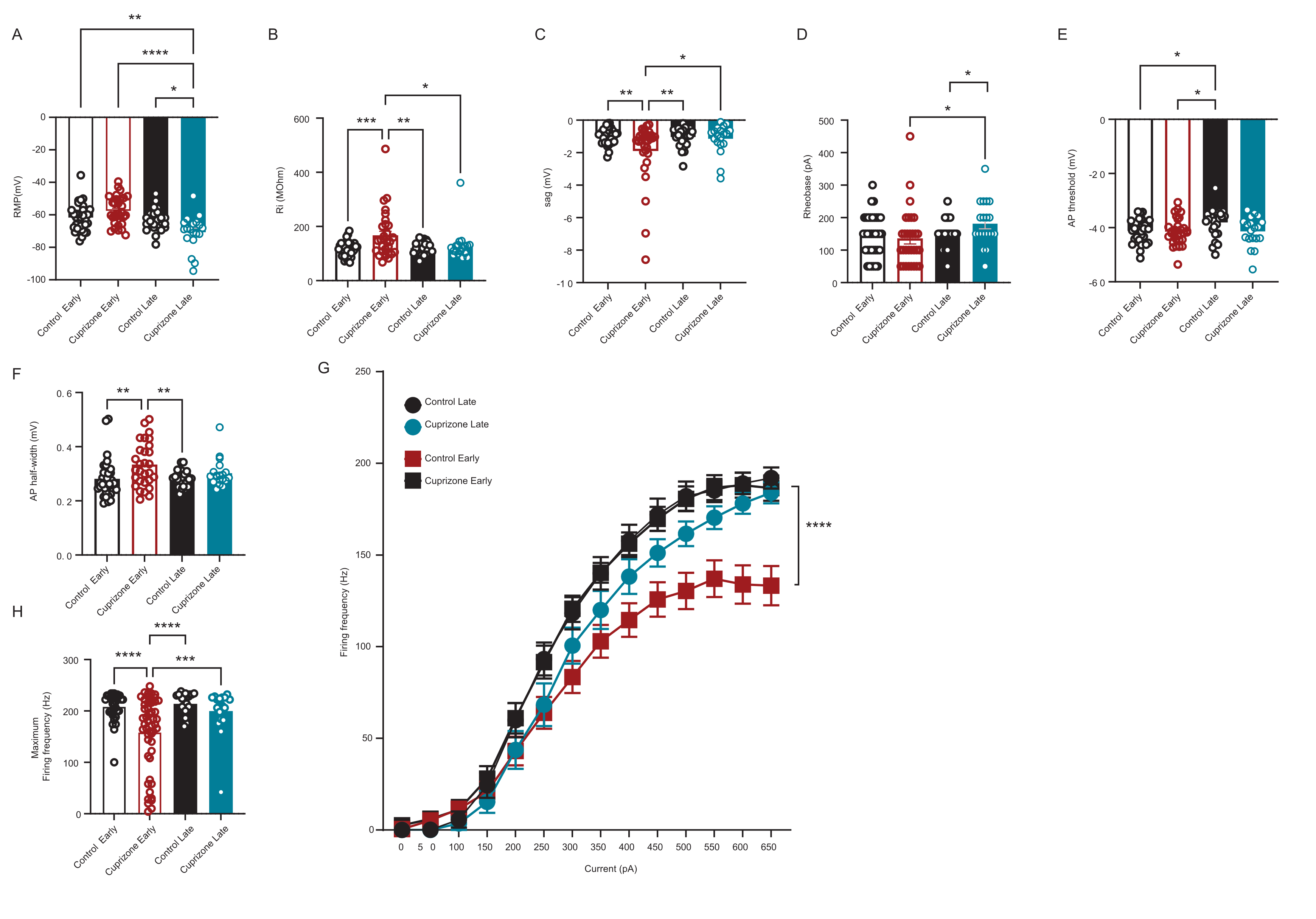

Supplement: S1 Fig — A–I. Juvenile demyelination leads to impairment in PV interneuron maturation whereas adult demyelination induces a decrease in the excitability of PV interneurons. Summary data showing the averaged (± s.e.m) of the following intrinsic properties: (A) Resting membrane potential (RMP) (ANOVA: n = 36,30,19,24 cells from 9/8/8/6 mice per group, ****p < 0.0001, post-hoc LSD test: *p < 0.05, **p < 0.01, ****p < 0.0001), (B) Input resistance (IR) (ANOVA: n = 36,30,19,24 cells from 9/8/8/6 mice per group, **p = 0.0024, post-hoc LSD test: *p < 0.05, **p < 0.01, ***p < 0.001), (C) Sag (ANOVA: n = 36,30,19,24 cells from 9/8/8/6 mice per group, **p = 0.0083, post-hoc LSD test: *p < 0.05, **p < 0.01), (D) Rheobase (ANOVA: n = 36,30,19,24 cells from 9/8/8/6 mice per group, p = 0.1230, post-hoc LSD test: *p < 0.05) and (E) Action potential (AP) threshold (ANOVA: n = 36,30,19,24 cells from 9/8/8/6 mice per group, *p = 0.0452, post-hoc LSD test: *p < 0.05), (F) AP half-width (ANOVA: n = 36,30,19,24 cells from 9/8/8/6 mice per group, **p = 0.0058, post-hoc LSD test: **p < 0.01), (G) Average action potential (AP) frequency in response to 0–650 pA current steps illustrating no significant change in S1 PV interneuron firing frequency following juvenile demyelination (group x current two-way repeated measures: n = 36,30,19,24 cells from 9/8/8/6 mice per group: F(39,1,291) = 2.487, ****p < 0.0001) and (H) Maximum firing frequency per group (ANOVA: n = 36,30,19,24 cells from 9/8/8/6 mice per group, ****p < 0.0001, post-hoc LSD test: ***p < 0.001, ****p < 0.0001). The data displayed in (A–F) and (H) can be found in S10 Table. (TIFF) [file pbio.3003421.s001.tiff]

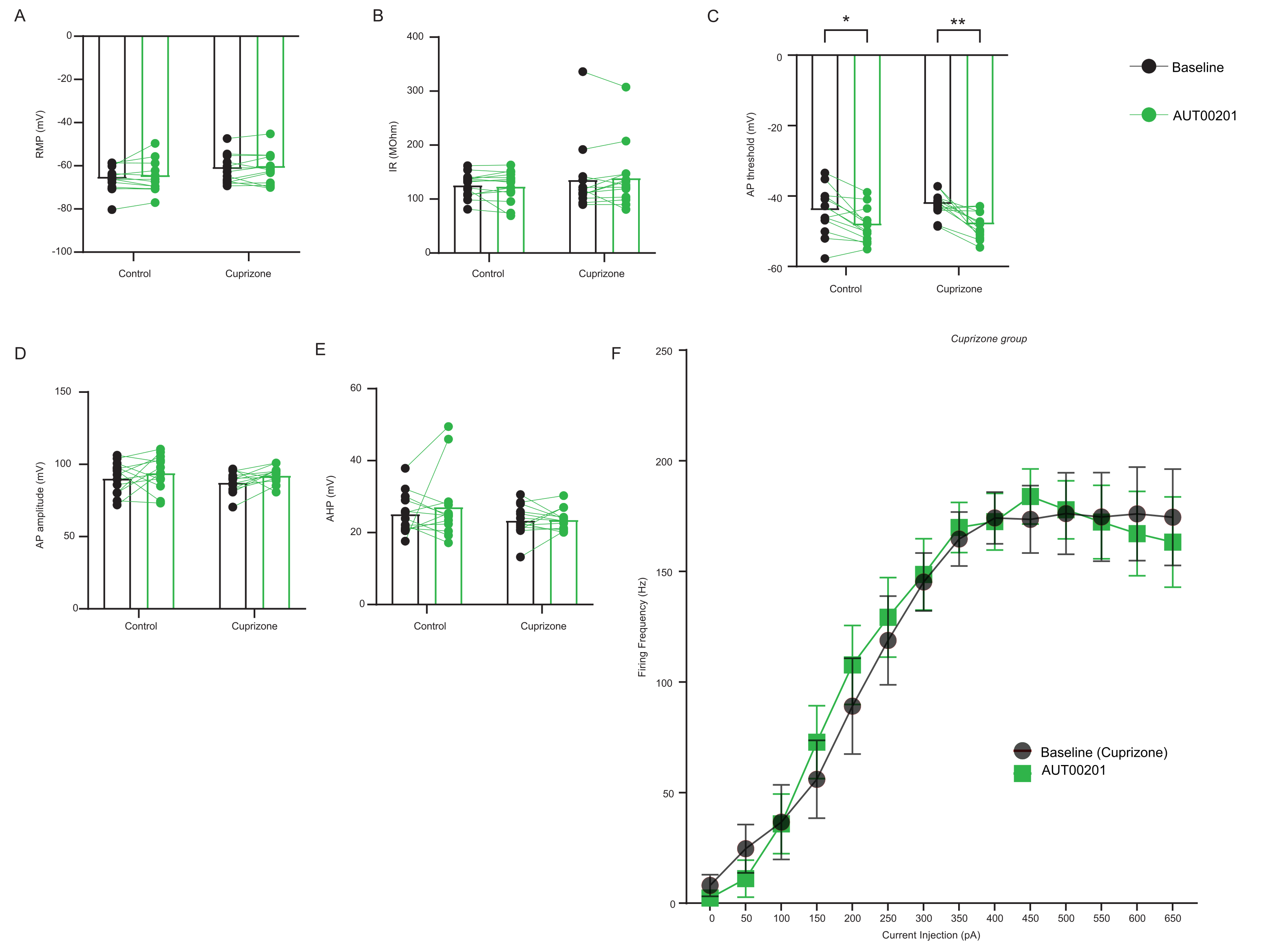

Supplement: S2 Fig — A-B. Bath application of AUT00201 (1 µM) had no significant effect on the resting membrane potential (A) or the input resistance (B) of PV interneurons from cuprizone mice and control mice (RMP: group x AUT00201 two-way repeated measures: n = 12,13 cells from 4 mice per group: p = 0.8593; AUT00201 effect: p = 0.2171. IR: group x AUT00201: p = 0.8605; AUT00201 effect: p = 0.4704). C–E. AUT00201 decreased the AP threshold of PV interneurons in both groups (group x AUT00201 two-way repeated measures: n = 12,13 cells from 4 mice per group: p = 0.4503; AUT00201 effect: ****p < 0.0001. Post-hoc LSD analysis: *p < 0.05, **p < 0.01) while not having an effect on either the AP amplitude (D) or the AHP (E) (D: group x AUT00201 two-way repeated measures: n = 12,13 cells from 4 mice per group: p = 0.8235; AUT00201 effect: p = 0.0763. E: group x AUT00201: p = 0.6088; AUT00201 effect: p = 0.3628. F. There was no effect detected on the overall firing frequency of PV interneurons from cuprizone-treated mice after bath application of AUT00201 when averaging all the cells together (group x current two-way repeated measures: n = 13 cells from 4 mice per group: F(13,78) = 0.9280, p = 0.5287. AUT00201 effect: F(1,6) = 5.05, p = 0.0657). The data displayed in (A–E) can be found in S10 Table. (TIFF) [file pbio.3003421.s002.tiff]

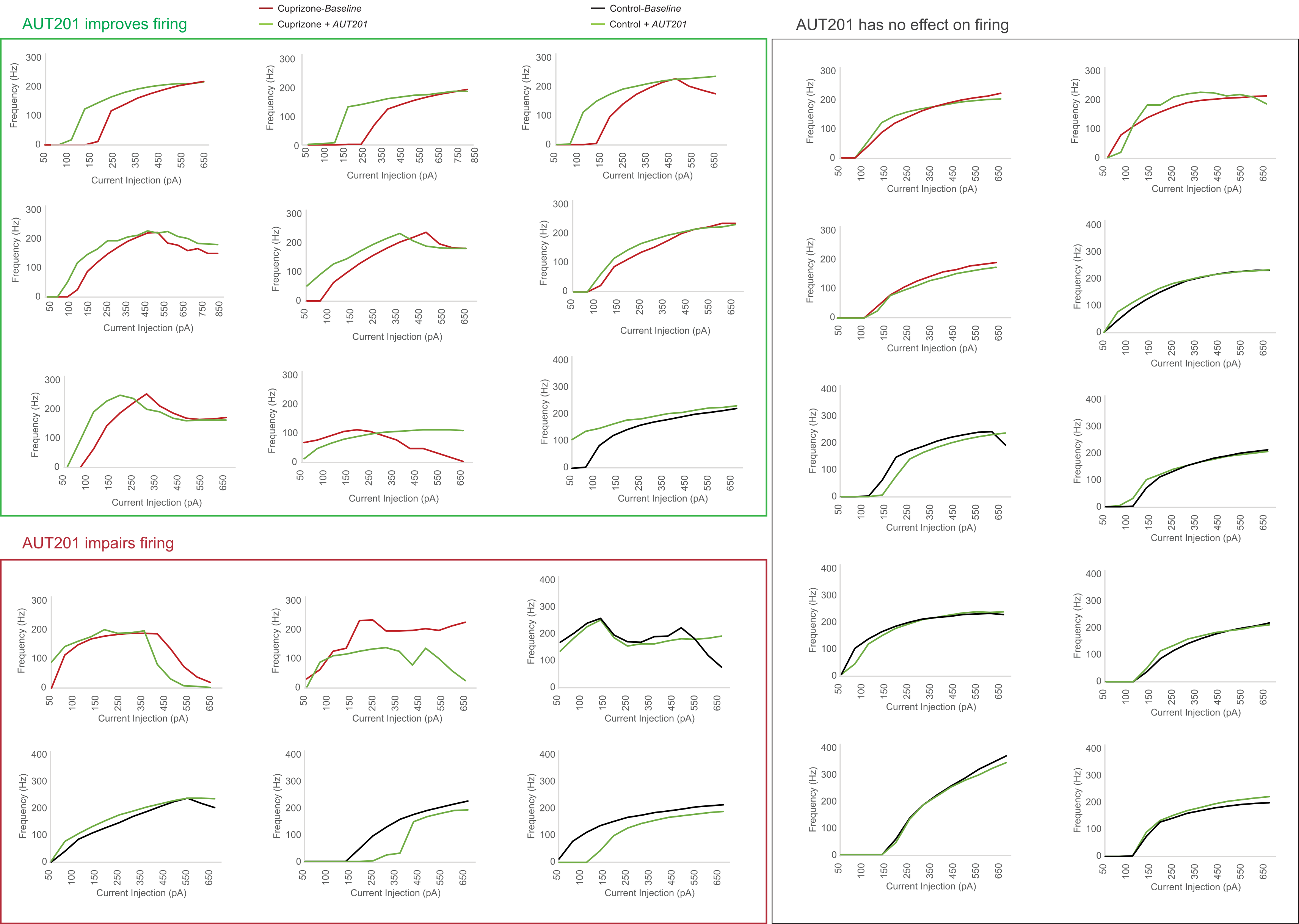

Supplement: S3 Fig — Effect of bath application of AUT00201 (1 µM) (green) on the firing frequency of individual PV interneurons in control (black) and cuprizone-treated (red) mice. The cells were divided into three groups: AUT00201 improved the firing, impaired the firing or had no effect. (TIFF) [file pbio.3003421.s003.tiff]

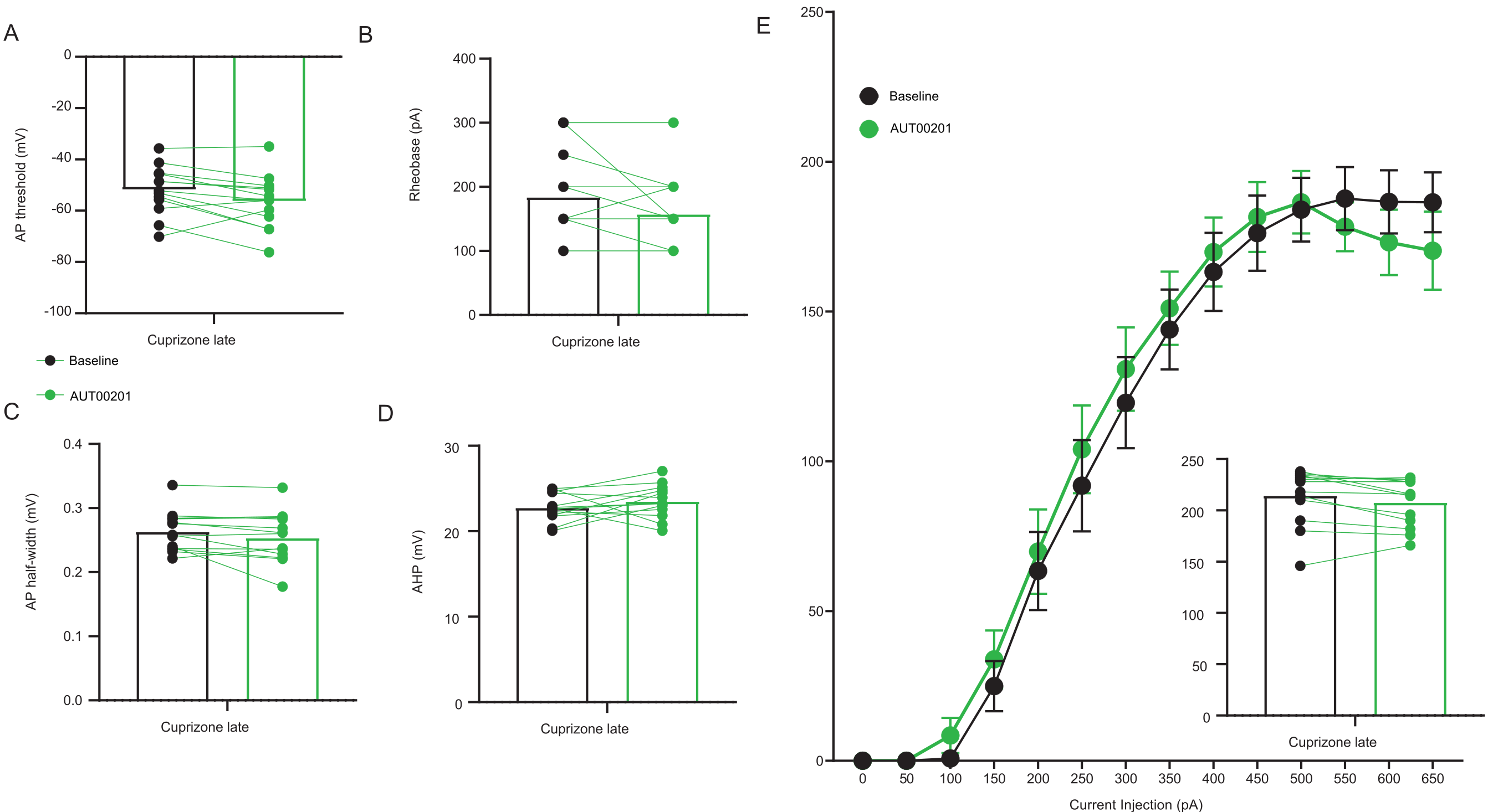

Supplement: S4 Fig — A–E. Bath application of AUT00201 (1 µM) had no significant effect on any of the intrinsic properties of PV interneurons following adult demyelination. Summary data showing the averaged (± s.e.m) of the AP threshold (A), the rheobase (B), the AP half-width (C), the AHP (D) and the overall firing frequency of PV interneurons from mice that underwent adult demyelination (E) (Paired t test: A: p = 0.1006, B: p = 0.0678, C: p = 0.1006, D: p = 0.2812, E: p = 0.0653; two-way repeated measures: AUT00201 effect: F(1,12) = 0.4990, p = 0.4934). The data displayed in (A–E) can be found in S10 Table. (TIFF) [file pbio.3003421.s004.tiff]

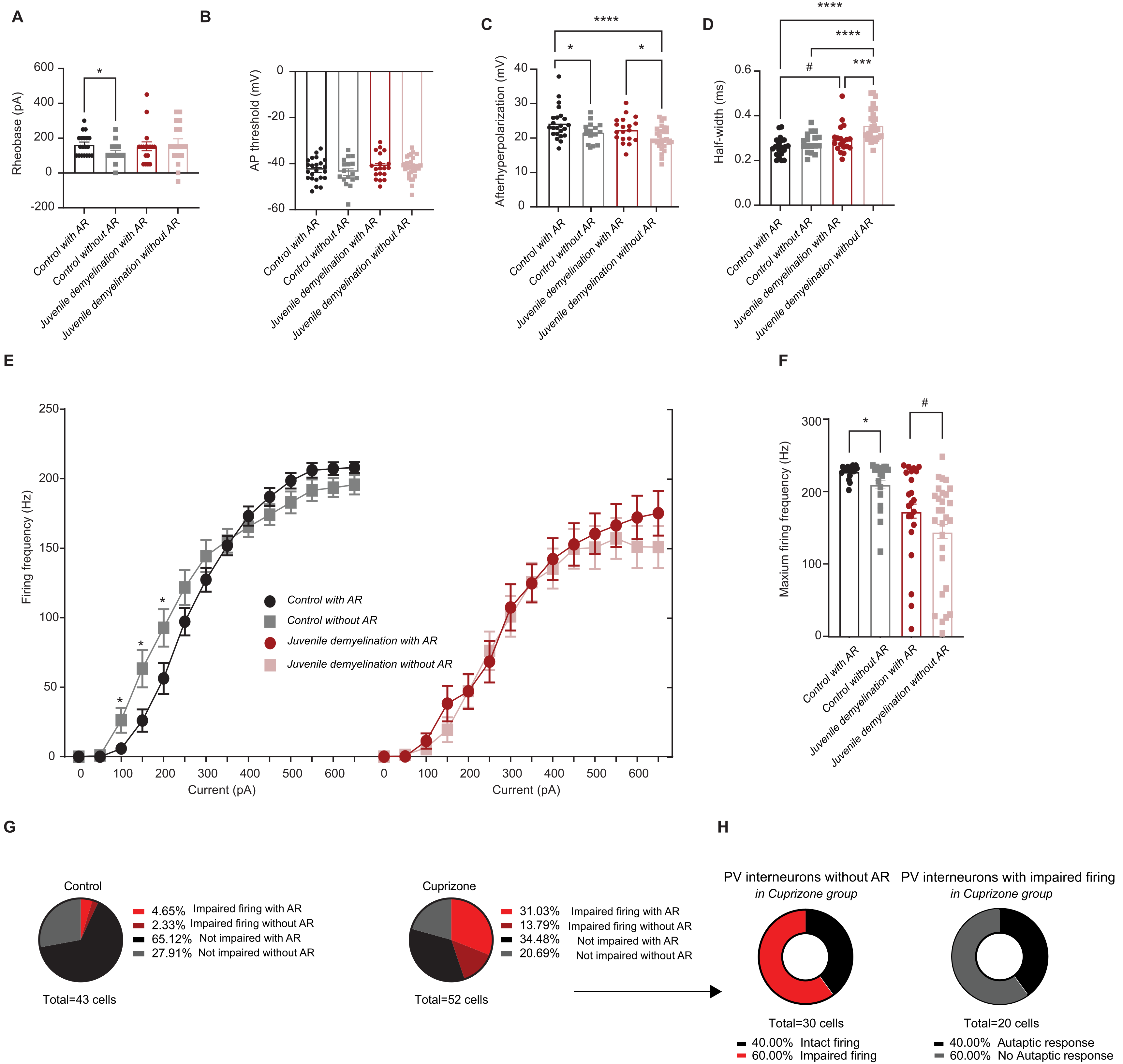

Supplement: S5 Fig — A–F. Summary data of the averaged (± s.e.m) altered intrinsic properties of PV interneurons grouped by whether they show autaptic responses or not in both the control and the cuprizone-treated group: (A) Rheobase (t test of control with vs. without autapse; n = 19 cells from 9 mice per group, *p < 0.01; t test of cuprizone with vs. without autapse; n = 17 cells from 8 mice per group, p = 0.6246), (B) Action potential (AP) threshold (t test of control with vs. without autapse; n = 19 cells from 9 mice per group, p = 0.5680; t test of cuprizone with vs. without autapse; n = 17 cells from 8 mice per group, p = 0.7648), (C) After-hyperpolarization (AHP) amplitude (ANOVA: n = 19,19,18,18 cells from 9−8 mice per group, p = 0.0010, post-hoc LSD test: *p < 0.05, ****p < 0.0001) and (D) AP half-width (ANOVA: n = 19,19,18,18 cells from 9−8 mice per group, p = 0.0002, post-hoc LSD test: #p = 0.0607 ***p < 0.001, ****p < 0.0001). E. Average action potential (AP) frequency in response to 0−650 pA current steps illustrating a significant increase in PV interneuron firing frequency at low-current steps in control cells without autapses (gray) compared to control cells with autapses (black). Note that in cuprizone mice there was no difference (group x current two-way repeated measures: n = 19,19,18,18 cells from 9−8 mice per group: F(39,856) = 1.671, p = 0.0068, post-hoc LSD test: *p < 0.05). F. Summary data of the maximum firing frequency per group revealing a decrease in the maximum firing frequency in PV interneurons that do not have autapses (t test of control with vs. without autapse; n = 19 cells from 9 mice per group, *p < 0.01; t test of cuprizone with vs. without autapse; n = 17 cells from 8 mice per group, p = 0.0722). G. Pie chart showing the fraction of cells with impaired or intact firing and with or without autapses in both control mice (left) or cuprizone-treated mice (right). H. Left. Fraction of cells with intact or impaired firing within all the cells that had no [file pbio.3003421.s005.tiff]
