# Supplementary material for: Transient juvenile demyelination impairs maturation and function of parvalbumin-positive interneurons in the prefrontal cortex
Source: PLoS Biol. 2025 Sep 30;23(9):e3003421. doi: 10.1371/journal.pbio.3003421 (PMC12510662; doi:10.1371/journal.pbio.3003421)
Supplement: Attachment — Submitted filename: Rebuttal PLOS Biology Final.docx [file pbio.3003421.s019.docx]

**Dear Dr. Taylor Hart,**

**Thank you for the opportunity to revise our manuscript and for allowing us to address the reviewers’ comments. We have carefully considered all the feedback and have responded to each concern in detail.**

**We believe that the manuscript has significantly improved as a result of the review process, and we sincerely appreciate the constructive input provided by the reviewers.**

**We hope that the revised version meets the standards of the journal and will be considered favorably for publication.**

**Sincerely,**

**On behalf of all co-authors**

**Point-by-point response to the reviewers’ and Editor’s comments:**

The comments of the reviewers are in regular text below, followed by our responses in *blue italics*. Within the revised manuscript, changes to the text compared to the originally submitted manuscript have been indicated using Track Changes.

**Reviewer #1:**
The manuscript "A critical period for prefrontal cortex PV interneuron myelination and maturation" by Hijazi and colleagues addresses a highly relevant question - is the myelination of PV interneurons in the murine prefrontal cortex subject to critical periods during development? The study proceeds to very comprehensively show that yes indeed, there seems to a very relevant developmental period that shapes myelination in this cell type. To reach this conclusion, the authors employ a number of carefully conducted experiments, with the ephys recordings being the most thorough and convincing. The experiments are well-designed, statistically sound, and provide all data necessary to reach the conclusions offered in this manuscript. I find this study of significant impact, novelty, and certainly of interest for a multidisciplinary readership.

> *We sincerely thank the reviewer for their careful and insightful evaluation of our manuscript. We are grateful for the positive remarks regarding the significance, novelty, and rigor of our study, as well as the thoroughness of the electrophysiological recordings and experimental design.*

My comments mostly concern the methods section, which in my opinion could profit from more detail on experimental procedures.
1. The methods do not outline how immunofluorescence (not immunohistochemistry!) was quantified, especially in Fig. 5 where this is crucial to understand the findings regarding Kv3.1 expression changes. Using fluorescence intensity as a quantitative measure is highly problematic (a semi-quantitative method at best, and if not run with internal controls, this is hard to interpret). Please provide details for your quantification approach, including how many ROIs were analyzed.

> *We thank the reviewer for this valuable comment and sincerely apologize for the oversight in omitting details of our immunofluorescence quantification methods. This section has now been updated in the Methods to clarify how Kv3.1 expression was analyzed. We have also added a new Key Resources Table to highlight all the reagents, chemicals and software used.*

*The following text has now been added to the Methods: “Images were acquired using a Zeiss LSM 700 confocal microscope (Carl Zeiss) equipped with Plan-Apochromat objectives: 10×/0.45 NA, 40×/1.3 NA (oil immersion), and 63×/1.4 NA (oil immersion). Alexa Fluor 488 and Cy3-conjugated secondary antibodies were visualized using excitation wavelengths of 488 nm and 555 nm, respectively. A 10× objective was used to capture low-magnification images, followed by corresponding high-resolution whole-cell images acquired with the 63× oil immersion objective using 555 nm excitation. Imaging parameters were standardized across all image acquisition, with the pinhole set to 0.2% and gain adjusted between 750–800 units to optimize signal-to-noise ratio. Biocytin-filled cells were imaged using tiled z-stacks (512 × 512 pixels) with a step size of 1 µm. Resectioned slices of these cells were imaged at 40× magnification using both 488 nm and 555 nm excitation wavelengths. Myelin basic protein (MBP) staining was visualized at 40× magnification (1024 × 1024 pixels) with 555 nm excitation. All imaging settings were kept consistent across samples to ensure uniform fluorescence quantification. For Kv3.1b immunofluorescence analysis, confocal images of PV and Kv3.1 labeling were taken using 40x/1.3 NA (oil immersion) objective. 20x images were used for quantification of cell numbers, and 40x z-stack confocal images were used for fluorescence analysis. Kv3 intensity was quantified as previously described ^1^. In brief, four stacks (same ROIs) of layer 2/3 PFC were quantified per mouse (3 mice per group). Selected cells were manually outlined, and fluorescence of individual cells was measured using the measure stack plugin in Fiji (ImageJ 5.12h). Using the raw images, ROIs were used to outline each cell and measure parameters including area, mean fluorescence, and integrated density, along with several background fluorescence readings from adjacent regions. The corrected total cell fluorescence was then calculated using the following formula: Integrated Density - (Area x Mean background fluorescence). For MBP immunofluorescence analysis, confocal tile (5x5) images of PFC layer 2/3 were taken using 40x/1.3 NA (oil immersion) objective. Four stacks were quantified per mouse. ROIs of identical sizes were manually outlined to cover layer 2-3 of PFC, and fluorescence intensity was measured using the measure stack plugin in Fiji (ImageJ 5.12h).”*

*We completely agree with the reviewer that fluorescence intensity is at best a semi-quantitative measure and not ideal for making definitive claims about expression changes, particularly without internal controls. Our goal with the immunofluorescence analyses was to provide supporting evidence, but we fully recognize its limitations. Accordingly, we have now modified the relevant text of the Discussion to better reflect this reality (lines 559-567).*

2. The methods chapter on immunohistochemistry (which should be corrected to immunofluorescence) does not contain even the barest of protocols, and only refers to previous papers. Please outline at least the minimum information so that a reader can evaluate the procedure without having to check other publications. You do this for your ephys recordings, ("in brief, ..."), please include this here as well. Which controls were run for the antibodies?

> *We fully agree and appreciate the opportunity to clarify this important point in the manuscript. As suggested, we have also replaced the term ‘immunohistochemistry’ to ‘immunofluorescence’, and included a more detailed description of our protocol in the Methods section. This includes fixation procedures, sectioning, blocking and permeabilization steps, primary and secondary antibody incubation details, and imaging parameters.:*

*“Mice were anaesthetized with intraperitoneal pentobarbital natrium (Nembutol®) prior to cardiac perfusion using 4 % formaldehyde (PFA). Brains were removed and fixed in 4% PFA for 2 h at room temperature. Next, brains were transferred into 10% sucrose phosphate buffer (0.1 M PB) and stored overnight at 4°C. To improve the slicing process, brains were embedded in 12% gelatin-10% sucrose blocks, incubated for 1.5 h at room temperature in 10% paraformaldehyde-30% sucrose solution (PB 0.1 M), and then transferred for overnight incubation at 4°C in 30% sucrose solution. Brains were subsequently sectioned coronally at 40 µm thickness using a freezing microtome (Leica, Wetzlar, Germany; SM 2000R) and stored in 0.1 M PB. For immunofluorescence labeling, slices were blocked with 0.5% Triton X-100% (MerkMillipore) and 10% normal horse serum (NHS; Invitrogen, Bleiswijk, The Netherlands) for 1 h at room temperature and incubated for 72 h at 4°C with primary antibodies [mouse anti-Kv3.1 (1:300, Synaptic systems, Cat. No.: 242 003), rabbit anti-PV (1:1000, Swant PV25), goat anti-MBP (1:300, Santa Cruz, C-16, sc-13 914)] in PBS buffer containing 0.4% Triton X-100 and 2% NHS. Secondary antibodies [anti-mouse Alexa 488 (1:300, Invitrogen), anti-rabbit Cy3 (1:300, Invitrogen) and anti-rabbit Alexa 647 (1:300, Invitrogen)] were applied for 2 h at room temperature after thorough washing. Slices were cover slipped with Vectashield H1000 fluorescent mounting medium (Vector Labs, Peterborough, UK). Recovery of biocytin-labelled cells following electrophysiological recordings was performed as previously described (Stedehouder et al. 2019). In brief, patch-clamp recorded PV interneurons were filled with 5mg/mL biocytin during whole-cell recordings, fixed with 4% (PFA) overnight, and stored in PBS at 4°C. Specifically, 300 µm slices were incubated overnight at 4°C in fresh 4% paraformaldehyde (PFA). Slices were extensively rinsed at room temperature in PBS and stained in PBS buffer containing 0.4% Triton X-100, 2% normal horse serum (NHS; Invitrogen, Bleiswijk, The Netherlands) and streptavidin-Cy3 (1:300; Invitrogen) overnight at 4°C. Slices were washed with PBS and PB 0.1 M and mounted on slides, cover slipped with 150 µl Vectashield, sealed, and imaged for their axonal morphology (see Confocal imaging and Reconstruction). To avoid excessive thinning or dehydration of 300 µm sections, cells were mounted, immediately imaged, and returned to PB 0.1 M immediately after imaging.*

*After full cell imaging, 300 µm slices were extensively washed in PB 0.1 M and incubated overnight at 4°C in 30% sucrose (0.1 M PB). Sections were then carefully recut at 40 μm using a freezing microtome (Leica, Wetzlar, Germany; SM 2000R) and stored serially in 0.1 M PB at 4°C. Serial 40 µm sections were extensively washed with PBS and pre-incubated with a blocking PBS buffer containing 0.5% Triton X-100% and 10% NHS for 1 hr at room temperature. Sections were incubated in PBS buffer containing 0.4% Triton X-100% and 2% NHS for 72 hr at 4°C and primary antibodies [mouse anti-PV (1:1000, Swant, 235), and goat anti-MBP (1:300, Santa Cruz, C-16, sc-13 914)]. Next, sections were washed with PBS and incubated with corresponding Alexa-conjugated secondary antibodies (1:300, Invitrogen) in PBS buffer containing 0.4% Triton X-100, 2% NHS for 5 hr at room temperature. Subsequently, sections were washed with PB 0.1M, mounted onto slides, cover slipped with Vectashield H1000 fluorescent mounting medium (Vector Labs, Peterborough, UK), sealed, and imaged.”*

*We have also added a new Key Resources Table to highlight all the reagents, chemicals and software used (Supplementary Table 1).*

3. The methods chapter on confocal imaging is missing important information on the objective (NA, immersion media?), and the laser lines used with this microscope. Again, please include at least a brief overview of your reconstruction procedure. If your step size for imaging z-stack was 1 µm, applying Nyquist conditions, you cannot adequately quantify structures smaller than 1 µm. Since this was apparently already done for previous publications (quantification of autapses), maybe the authors can elaborate a bit on how this important morphological detail was quantified. Maybe in the figure on autapses and their morphology, the authors could include a high res insert that shows what these putative contact points between dendrite and axon look like? It is impossible to tell from Fig. 7E as it stands now.

> *We again concur with the reviewer. Accordingly, the methods section has now been extended to provide much greater detail:*

*“Overview images were imported into Neurolucida 360 software (v2.8; MBF Bioscience) for axonal reconstruction using the interactive tracing method with Directional Kernels. Axons were classified as myelinated if at least one MBP-positive internode was present, and as unmyelinated if no MBP-positive segments were observed up to at least the 10th branch order. Images were taken using a Zeiss LSM 700 microscope. Biocytin-filled whole-cell overview images were acquired using a 63x magnification objective with tiled z-stack images (512 × 512 pixels) and a step size of 1 μm. Three-dimensional cell reconstruction was performed using Neurolucida software (MBF Bioscience) as previously described (Stedehouder et al. 2017; Stedehouder et al. 2019). Structural autapses were operationally defined as individual locations In which the distance between between an axonal bouton and a dendritic spine or stem of the same cell was less than 1 μm. This was automatically detected using Neuroleucida after full reconstruction of the cell and identification of axons and dendrites. Image analysis was strictly performed with the experimenter blind to experimental group and functional autaptic connectivity.”*

*We have also added a representative image in Figure 7E of a filled cell including arrowheads indicating the axodendritic point of contact, defined as a structural autapse. Moreover, we have also included a supplementary video (Supp. video 1) demonstrating a representative Neurolucida reconstruction with structural autapses highlighted in red.*

4. A more accurate statistical depiction would be to include STDEV instead of SEM, since you show individual cells as n, and not grouped across animals. The authors may want to reconsider this, but I leave it up to them.

> *We thank the reviewer for this thoughtful suggestion and agree that standard deviation (STDEV) may more directly reflect variability when individual cells are used as the unit of analysis. However, since all of our figures display individual data points along with the mean ± SEM, we have opted to retain SEM; but we appreciate the reviewer raising this issue and have clarified it in the figure legends where appropriate.*

Discussion
The last chapter titled "Relevance for human disorders" is a bit confusing since it is not clear right away, which of the references are work done in rodent or human. This is also something I noticed while reading the otherwise very helpful introduction (paragraph starting with "Abberant PV interneuron maturation …."). Maybe the authors could clarify which species is investigated in their references choices?

> *We agree, and thank the reviewer for this observation. Accordingly, we have now revised the “Relevance for human disorders” section, as well as the relevant paragraph in the Introduction, to clearly indicate the species used in each referenced study.*

As a general suggestion - the authors may consider to switch the color scheme of all red/green figures to one that is more suitable for color vision-deficient readers. The red and green is very hard to discern, especially in those images offering overviews.

> *We appreciate this important suggestion and have revised Figure 1, Figure2, and Supp. Figure 2 to replace the prior red/green color scheme with a more accessible red/grey combination, which improves visibility for color vision-deficient readers. We will continue to keep accessibility in mind with future manuscripts as well, and thank the reviewer for highlighting this issue.*

**Reviewer #2:**

This paper studies the role of myelin on PV-neuron maturation during adolescence, a critical developmental period for PFC maturation. The results show that demyelination during the juvenile period, when the PFC and PV interneurons undergo maturation, leads to changes in PV morphology and electrophysiological properties. Specifically, cuprizone-induced demyelination is associated with compromised high-frequency firing, decrease in Kv3 currents and disrupted autaptic inhibition. These effects were only partly rescued with remyelination. Conversely, demyelination in adulthood was not associated with similar changes. Overall, this manuscript makes a significant contribution to the field and provides compelling evidence that demyelination can play a crucial role in PV-circuitry and PFC maturation, with potential implicatios to psychiatric disorders. The manusrcipt is very well-written and the conclusions of this paper are generally well-supported by the data.

> *We genuinely thank the reviewer for their detailed and constructive comments on our manuscript. We appreciate the recognition of the significance of our findings and the clarity of our writing. These constructive comments and suggestions have helped to further strengthen the manuscript.*

However, the following points require clarification.
a) The authors state that: "Juvenile cuprizone treatment led to a clear decrease in myelination in adulthood and a complete demyelination of PV interneuron axons in the PFC". Was the loss of myelin, or remyelination following cuprizone, on PV-neurons quantified somehow? Since the effects of cuprizone on demyelination can be dependent on age, strain, brain region etc, quantification of the de/remyelination would help to rule out the possibility that some of the changes are due to incomplete de/remyelination. Or if the effects of cuprizone on PV-myelination in juvenile mice are characterized elsewhere, please provide reference to it.

> *We thank the reviewer for raising this important point. We have now added a quantification of myelin (MBP) levels in layer 2/3 of the prefrontal cortex, which confirms a significant reduction in myelin in the juvenile demyelination group and a return to baseline levels following the remyelination phase, as shown below (from Figure 1D and 9B). These analyses have also been added to the Results and Methods sections in the revised manuscript.*

*In addition, we confirm that none of the reconstructed PV interneurons from the juvenile demyelination group were myelinated (0/7 cells). However, we also acknowledge that our current approach does not allow for a sensitive or accurate quantification of remyelination at the single-cell level. While the data we have acquired suggests a substantial degree of remyelination, we recognize that this may not fully reflect the original pattern or extent of myelination. This limitation is especially relevant given recent reports showing that remyelination is often incomplete and does not restore the original topographical pattern of myelination, even after extended recovery periods (e.g., 8 weeks or more)*^2,3^*.Accordingly, we now discuss this limitation in the revised manuscript and agree that more precise methods would be valuable to accurately resolve the degree and topography of remyelination in future studies (lines 635-645).*

b) Given the extensive, and highly relevant, discussion regarding the effects of social isolation stress on PFC myelination, I assume the mice in the experiment were group housed to rule out the effects of social isolation on control and cuprizone mice. This should be clearly indicated in the materials and methods.

> *We thank the reviewer for this important observation and appreciate the opportunity to better clarify this detail. All mice were indeed group housed throughout the experiment to avoid confounding effects of social isolation on PFC myelination. This information has now been explicitly added to the Materials and Methods.*

c) The experiments included both male and female mice. Did the authors evaluate whether there were any sex-specific effects? If sex differences were not assessed (either due to small sample sizes or other limitations), or if no differencies were present, this should be explicitly stated.

> *We thank the reviewer for raising this important point. In response, we have now re-analyzed our electrophysiological data independenly by the sex of the animals (see figure panels below). While the overall trends and results are comparable between male and female mice, we acknowledge that our sample size—once split by sex—is likely underpowered to detect more subtle or nuanced differences. Accordingly, we have now modified the Discussion section noting that while sex-specific differences of large effect can be excluded by our current dataset, we cannot rule out the presence of more subtle differences (lines 688-692).*

d) Kv3 immunofluorescence expression experiment is missing from the materials and methods. Was the intensity measured on the soma and/or along the axons of PV-neuron? While the electrophysiological experiments show solid evidence for altered Kv3 conduction in the model, the discussion could benefit from a short section where it is discussed how lack of myelin could lead to reduced Kv3 expression (especially if the analysis is focused on the channels on the soma).

> *We thank the reviewer for this valuable comment and sincerely apologize for the oversight in omitting details of our immunofluorescence quantification methods. This section has now been updated in the Methods to clarify how Kv3.1 expression was analyzed. We have also added a new Key Resources Table to highlight all the reagents, chemicals and software used.*

*The following text has now been added to the Methods:“Images were acquired using a Zeiss LSM 700 confocal microscope (Carl Zeiss) equipped with Plan-Apochromat objectives: 10×/0.45 NA, 40×/1.3 NA (oil immersion), and 63×/1.4 NA (oil immersion). Alexa Fluor 488 and Cy3-conjugated secondary antibodies were visualized using excitation wavelengths of 488 nm and 555 nm, respectively. A 10× objective was used to capture low-magnification images, followed by correspondinghigh-resolution whole-cell images acquired with the 63× oil immersion objective using 555 nm excitation. Imaging parameters were standardized across all image acquisition, with the pinhole set to 0.2% and gain adjusted between 750–800 units to optimize signal-to-noise ratio. Biocytin-filled cells were imaged using tiled z-stacks (512 × 512 pixels) with a step size of 1 µm. Resectioned slices of these cells were imaged at 40× magnification using both 488 nm and 555 nm excitation wavelengths. Myelin basic protein (MBP) staining was visualized at 40× magnification (1024 × 1024 pixels) with 555 nm excitation. All imaging settings were kept consistent across samples to ensure uniform fluorescence quantification. For Kv3.1b immunofluorescence analysis, confocal images of PV and Kv3.1 labeling were taken using 40x/1.3 NA (oil immersion) objective. 20x images were used for quantification of cell numbers, and 40x z-stack confocal images were used for fluorescence analysis. Kv3 intensity was quantified as previously described ^1^. In brief, four stacks (same ROIs) of layer 2/3 PFC were quantified per mouse (3 mice per group). Selected cells were manually outlined, and fluorescence of individual cells was measured using the measure stack plugin in Fiji (ImageJ 5.12h). Using the raw images, ROIs were used to outline each cell and measure parameters including area, mean fluorescence, and integrated density, along with several background fluorescence readings from adjacent regions. The corrected total cell fluorescence was then calculated using the following formula: Integrated Density - (Area x Mean background fluorescence). For MBP immunofluorescence analysis, confocal tile (5x5) images of PFC layer 2/3 were taken using 40x/1.3 NA (oil immersion) objective. Four stacks were quantified per mouse. ROIs of identical sizes were manually outlined to cover layer 2-3 of PFC, and fluorescence intensity was measured using the measure stack plugin in Fiji (ImageJ 5.12h).”*

*We completely agree with the reviewer that fluorescence intensity is at best a semi-quantitative measure and not ideal for making definitive claims about expression changes, particularly without internal controls. Our goal with the immunofluorescence analyses was to provide supporting evidence, but we fully recognize its limitations. Accordingly, we have now modified the relevant text of the Discussion to better reflect this reality (lines 559-567).*

*We have now also added a short paragraph to the Discussion addressing potential mechanisms by which loss of myelin could lead to reduced Kv3 expression (lines 544-558). This includes consideration of activity-dependent signalling, metabolic support from oligodendrocytes, and altered local ion homeostasis—all of which may influence ion channel expression and stability. We agree this provides important context for our findings.*

e) The absence of selectivity for specific axons or cell types leaves open the question of whether the observed changes are driven by demyelination of PV+ axons, loss of myelin in excitatory axons, or a combination of both. This limitation should be mentioned in the discussion.

> *We fully agree with the reviewer and have now added this important point to the discussion. As noted, our model does not allow us to selectively target demyelination in specific cell types, leaving open the possibility that the observed effects may result from myelin loss in PV interneuron’s axons, excitatory axons, or both. We now acknowledge this as a limitation in the Discussion and highlight the need for future studies using cell-type-specific approaches to disentangle their relative potential contributions to the observed effects (lines 688-704).*

Minor issues:
References 16-18 in the introduction shown as number and not written out as the other references.

Some of part of the figures seem to be cut out, Figure 1. text of the lower panels.
And in Figure 1 experimental timeline: change regular show to regular chow.

> *Thank you for noticing this issue, which we have now corrected.*

**Reviewer #3:**

This excellent paper breaks important new ground on the role of myelin in regulating the physiology of PV+ interneurons in neocortex.  It also dents, but doesn't kill one of my favorite hypotheses on the interaction between axon length and myelination of firing patterns in these neurons in this area (all the better!).  The paper shows a developmental critical period for neuronal plasticity of ion channel number/distribution, leading to significant alterations in the firing patterns of these neurons thought to be critical for a variety to animal behavior. 

The study uses the well-established cuprazone model for producing significant demyelination when added to diet.  It is demonstrated that demyelination during the period from P21 in mice, produces significant changes in the electrophysiological properties of PV neurons, that alter their repetitive firing patters; those patterns known to be important in behavior outcomes for the animal.  Among these changes are more hyperpolarized resting potentials, decreased current sags, decreased reobase, wider action potentials, lower frequency of action potential output, particular with high strength stimulation; in particular a decrease in maximum firing frequency and less ability to sustain repetitive firing.  A interesting finding is that much of this may be attributed to downregulation of KV3 potassium channels, an effect that can be partially rescued by upregulation by a positive modulator of the channel.  They also demonstrate morphological alteration of PV neuron axons, with shorter axons, and most interesting, fewer autapses.  These changes could be partially reversed when the neurons are allowed to remyelinate, adding additional confidence in their interpretation of the results.

Most surprising to me, was that adult demyelination produces few of the physiological changes, establishing the nature of the changes as happening within a critical developmental period.  I'm surprised that there was so little change in firing frequency, given the timing alterations in action potential arrival that would be presumed to occur with even adult demyelination.  But the results are clear that they isn't.

It would have been nice if they'd actually been able to measure any changes in conduction velocity with demyelination, but I'm not asking for this in the paper, because it is somewhat outside the scope of their work, and the techniques they employed were not capable of measuring it (this would require paired cell recording). 

I only have minor comments, most of which I leave to the discretion of the authors to address, although I think they would improve their paper (i.e. none of these are damaging to their data, or the interpretation of same).

> *We sincerely thank the reviewer for their generous and insightful evaluation of our work. We greatly appreciate the positive reception of our findings and the recognition of the novel contributions our study to understanding PV interneuron physiology and developmental myelination. These comments and constructive suggestions on the manuscript and related hypotheses are deeply appreciated and have further strengthened the revised manuscript.*

 1.  I can't find any mention of the species used, although there are clues ;). The species should be added to the title, the abstract, and species/strain to the M&M.

> *We fully agree and appreciate the reviewer’s suggestion, which we have now included to clearly indicate that we used mice. Moreover, in a similar regard, we have also taken greater care throughout the manuscript of indicating which species was used in studies we have cited.*

 2.  Even though the cuprazone model is well established in the field as a selective demyelinating agent, I think the interpretation of their results might be enhanced by some discussion of potential off-target effects, a worry, since the substance is administered systemically, and is known to have effect on peripheral organs, in particular the liver, which could, in turn, produce secondary metabolic effects that might produce some of the effects in brain that they describe. In this context, I'm particularly worried about the down regulation of KV3, which might simply be a correlated effect not caused by the loss of myelin.   I don't see this as any kind of fatal flaw, because this is the case with many if not most pharmacological manipulations, I just think it deserves a bit more discussion

> *We are in full agreement and appreciate the opportunity to clarify this important comment in the manuscript. Accordingly, we have now added* ***a limitation section*** *to our study that conveys the above-mentioned issues regarding cuprizone (lines 688-704).*

 3.  TEA is not all that selective for KV3, but the results are entirely consistent with the author's hypothesis.  Again, maybe just some additional discussion around this issue.

> *We appreciate the reviewer’s highly valid and important point. Accordingly, we have now added a brief discussion addressing the selectivity of TEA for Kv3 channels (lines 559-567). While our electrophysiological findings are consistent with altered Kv3 function, we fully acknowledge that TEA can affect other potassium channels, and this caveat is now explicitly stated to provide a balanced interpretation of the results.*
 
4.  There are a couple of places in the paper where the authors seem to imply that correlation proves causation, which of course, it doesn't.  The one that sticks out, in addition to the one listed in point 3, is the statement that loss of autapses and the changes in autapse facilitation, 'predicts' impairments in high frequency firing, when those factors only correlate with the changes in firing.

> *We appreciate the opportunity to avoid any such misunderstandings and have revised the manuscript to clarify this distinction. Of note, our intent with the use of the term ‘predicts’ was precisely to avoid implying a causal relationship. However, the reviewer’s impression convinces us that our use of this terminology has led to confusion, and therefore we have modifed the text accordingly, in an effort to avoid misinterpretations with regard to causality.*

5.  The most obvious explanation for the only partial recovery of physiologcal properties upon remyelination, is that recovery of myelin is also partial.  I would have made a stronger argument if they had an independent means of measuring the extent of myelination, which is something that is in the capability of the approaches utilized.

> *We thank the reviewer for raising this important point. We have now added a quantification of myelin levels in layer 2/3 of the prefrontal cortex, which confirms a significant reduction in myelin in the juvenile demyelination group and a return to baseline levels following the remyelination phase. These data have been added to the Results and Methods sections.*

*However, we acknowledge that our current approach does not allow for a sensitive or accurate quantification of remyelination at the single-cell level. While the data we have acquired suggests a substantial degree of remyelination, we recognize that this may not fully reflect the original pattern or extent of myelination. This limitation is especially relevant given recent reports showing that remyelination is often incomplete and does not restore the original topographical pattern of myelination, even after extended recovery periods (e.g., 8 weeks or more).*

*We now discuss this limitation in the revised manuscript (lines 635-645) and agree that more precise methods would be valuable to accurately resolve the degree and topography of remyelination in future studies.*

 6.  I think that the discussion, particularly the last paragraph, would be enhanced, by inclusion of some discussion of Karl Deiserroth's papers on the effects of altering PV neuron activity on animal behavior

> *We thank the reviewer for this valuable suggestion and fully agree. Accordingly, we have now incorporated a discussion of prior work involving targeted manipulations of PV neuron activity, and the consequent impact on animal behavior, in the final paragraph of the Discussion (lines 665-684), to better contextualize our findings within the broader literature.*

**References:**

1.	Stevens, S. R. *et al.* Ankyrin-R regulates fast-spiking interneuron excitability through perineuronal nets and Kv3.1b K+ channels. *eLife* **10**, (2021).

2.	Orthmann-Murphy, J. *et al.* Remyelination alters the pattern of myelin in the cerebral cortex. *eLife* **9**, (2020).

3.	Zirngibl, M., Assinck, P., Sizov, A., Caprariello, A. V. & Plemel, J. R. Oligodendrocyte death and myelin loss in the cuprizone model: an updated overview of the intrinsic and extrinsic causes of cuprizone demyelination. *Mol. Neurodegener.* **17**, (2022).
